# Carbon monoxide in an extremely metal-poor galaxy

**DOI:** 10.1038/ncomms13789

**Published:** 2016-12-09

**Authors:** Yong Shi, Junzhi Wang, Zhi-Yu Zhang, Yu Gao, Cai-Na Hao, Xiao-Yang Xia, Qiusheng Gu

**Affiliations:** 1 School of Astronomy and Space Science, Nanjing University, Nanjing 210093, China; 2 Key Laboratory of Modern Astronomy and Astrophysics (Nanjing University), Ministry of Education, Nanjing 210093, China; 3 Collaborative Innovation Center of Modern Astronomy and Space Exploration, Nanjing 210093, China; 4 Shanghai Astronomical Observatory, Chinese Academy of Sciences, 80 Nandan Road, Shanghai 200030, China; 5 Key Laboratory of Radio Astronomy, Chinese Academy of Sciences, Nanjing 210008, China; 6 Institute for Astronomy, University of Edinburgh, Royal Observatory, Blackford Hill, Edinburgh EH9 3HJ, UK; 7 ESO, Karl-Schwarzschild-Strasse 2, Garching 85748, Germany; 8 Purple Mountain Observatory, Chinese Academy of Sciences, 2 West Beijing Road, Nanjing 210008, China; 9 Tianjin Astrophysics Center, Tianjin Normal University, Tianjin 300387, China

## Abstract

Extremely metal-poor galaxies with metallicity below 10% of the solar value in the local universe are the best analogues to investigating the interstellar medium at a quasi-primitive environment in the early universe. In spite of the ongoing formation of stars in these galaxies, the presence of molecular gas (which is known to provide the material reservoir for star formation in galaxies such as our Milky Way) remains unclear. Here we report the detection of carbon monoxide (CO), the primary tracer of molecular gas, in a galaxy with 7% solar metallicity, with additional detections in two galaxies at higher metallicities. Such detections offer direct evidence for the existence of molecular gas in these galaxies that contain few metals. Using archived infrared data, it is shown that the molecular gas mass per CO luminosity at extremely low metallicity is approximately one-thousand times the Milky Way value.

## Abstract (web summary)

Extremely metal-poor galaxies in the local universe are the best analogues to investigating the interstellar medium at a quasi-primitive environment in the early universe. Here, the authors detect CO emission in a galaxy at 7% solar metallicity, offering direct evidence for the presence of molecular gas.

Galaxies in the early universe contained few metals (elements heavier than helium) and dust grains^1^. On the surface of dust grains, hydrogen atoms combined efficiently into hydrogen molecules^2^, which served as the fuel of star formation in present-day spiral galaxies, including our own Milky Way galaxy^3^. The lack of metals thus poses a question regarding the presence of molecular gas in the primordial galaxies through, for example, the gas-phase reaction^4^. The extremely metal-poor galaxies in the local universe, with the oxygen abundance relative to hydrogen <10% of the solar value, provide the best local insights into understanding the interstellar medium in a quasi-primitive environment. Although there is an indirect evidence for the presence of molecular gas in these galaxies^5^,^6^,^7^, the emission from the molecule carbon monoxide (CO), which is the primary tracer of molecular gas, has never been detected in them^8^,^9^,^10^,^11^,^12^,^13^.

In this study, we report the detection of CO in a galaxy at 7% of solar metallicity, along with additional detections in galaxies at 13% and 18% solar metallicity; these data offer direct evidence for the existence of molecular gas in these metal-poor galaxies. By comparing this data to the gas mass as traced by dust emission, the molecular gas mass per CO luminosity in these galaxies is found to be much higher than that of the Milky Way galaxy.

## Results

### Observations

**Figure 1:**
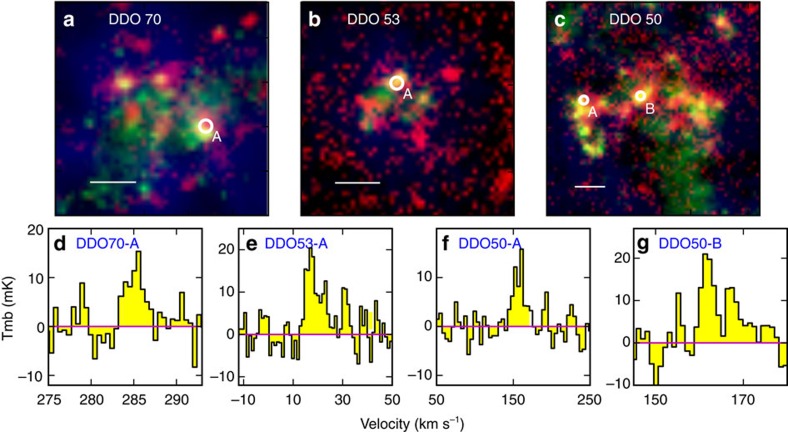
**False-colour images of three galaxies along with the CO** *J***=2−1 spectra**. (**a**) The image of DDO 70, where red denotes infrared emission at 160 μm, green denotes the far-UV emission and blue denotes the atomic hydrogen 21 cm emission. (**b**) The image of DDO 53. (**c**) The image of DDO 50. All the white scale bars are 40″ across. (**d**) The CO *J*=2−1 spectra for region A in DDO 70. (**e**) The CO *J*=2−1 spectra for region A in DDO 53. (**f**) The CO *J*=2−1 spectra for region A in DDO 50. (**g**) The CO *J*=2−1 spectra for region B in DDO 50. The spectral channel width is 0.5, 1.0, 4.0 and 1.0 km s^−1^ for DDO70-A, DDO53-A, DDO50-A and DDO50-B, respectively, and the corresponding 1−*σ* continuum sensitivity is 3.94, 4.17, 3.13 and 4.89 mK, respectively.

The galaxy DDO 70 is an extremely metal-poor galaxy at a distance of 1.38 Mpc (ref. 14), with the gas-phase oxygen abundance relative to hydrogen 12+log(O/H)=7.53 (ref. 15), compared with the solar abundance at 12+log(O/H)=8.66 (ref. 16). We have observed two additional dwarf galaxies at somewhat higher metallicity, including DDO 53 and DDO 50 at 3.68 Mpc (ref. 14) and 3.27 Mpc (ref. 14), respectively, with 12+log(O/H)=7.82 (ref. 17) and 7.92 (ref. 17), respectively. As shown in Fig. 1, we targeted four dusty star-forming regions in these three galaxies as listed in Table 1, using the Institut de Radioastronomie Millimetrique (IRAM) 30-m telescope. For each star-forming region, we pointed the telescope to the far-infrared peak that traces gas density enhancement with ongoing star formation. No prior CO detections of these regions have been reported, possibly because previous works targeted the peak of atomic gas often with a short exposure time^10^.

### CO emission

We detected CO *J*=2−1 emission in all four star-forming regions, including one in DDO 70 at 7% (labelled as DDO70-A), one in DDO 53 at 14% (labelled as DDO53-A) and two in DDO 50 at 18% (labelled as DDO50-A and DDO50-B) solar metallicity. The spectra and results are shown in Fig. 1 and listed in Table 1. The 1−*σ* continuum sensitivity is 3.94, 4.17, 3.13 and 4.89 mK for DDO70-A, DDO53-A, DDO50-A and DDO50-B, respectively, at a spectral resolution of 0.5, 1.0, 4.0 and 1.0 km s^−1^, respectively. The CO *J*=2−1 of DDO70-A has a S/N of 5.5 with a full width at half maximum (FWHM) of 2.4 km s^−1^, and the CO *J*=2−1 of DDO53-A is detected at a signal-to-noise ratio (S/N) of 7.1 with a FWHM of 7 km s^−1^. The CO line of DDO50-A has an integrated S/N of 5.9 with a FWHM of 18 km s^−1^. The emission of DDO50-B appears to have two velocity components. A single Gaussian fitting gives a value of S/N of 6.1 for the integrated strength peaked at the velocity of 163 km s^−1^ with an FWHM of 10 km s^−1^, and two Gaussian fittings give S/Ns of 6.1 and 3.2 for the two components at 161 and 167 km s^−1^, respectively, with FWHMs of 3.2 and 3.4 km s^−1^, respectively. The CO *J*=1−0 transition was covered by our observation but was not detected. The 3−*σ* lower limits to the ratios CO(*J*=2−1)/CO(*J*=1−0) in the main-beam temperature are 1.9, 0.9, 1.5 and 0.9 for DDO70-A, DDO53-A, DDO50-A and DDO50-B, respectively. As the size of a CO-emitting region shrinks significantly at the low metallicity^11^, we assumed point sources for CO-emitting regions relative to our IRAM beam (∼100–200 pc). The above ratio is thus still consistent with the assumption that the CO emission is thermalized and optically thick.

**Figure 2:**
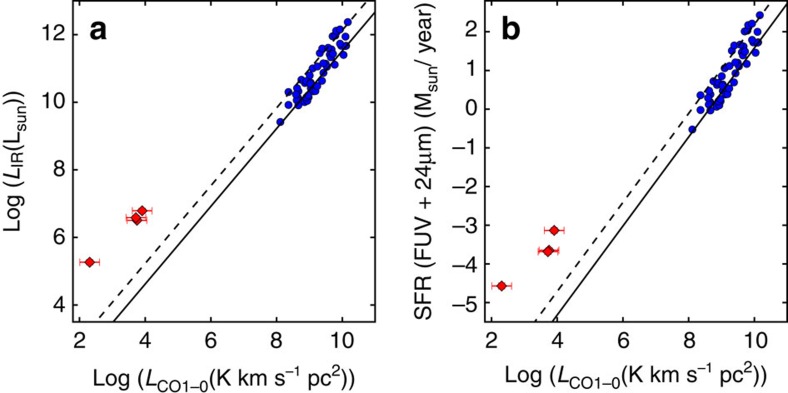
**The infrared luminosity and star-formation rate against CO luminosity**. (**a**) The infrared luminosity versus CO luminosity of our metal-poor star-forming regions compared with massive star-forming galaxies. The error bar is the s.d., which is basically the photon noise for each measurement of luminosity. (**b**) The SFR versus CO luminosity of our metal-poor star-forming regions as compared with massive star-forming galaxies. The error bar is the s.d. The error of the CO luminosity is the photon noise, and the error of the SFR is the photon noise plus systematic uncertainty. The red diamonds denote our observed four regions, and the blue circles denote massive star-forming galaxies^3^. The solid line is the best fit to star-forming disk galaxies^18^, whereas the dashed line is the best fit to the star-forming starburst galaxies^18^.

Figure 2 shows the total infrared luminosity (8–1,000 μm) versus the CO luminosity as well as the star-formation rate (SFR) versus the CO luminosity of these metal-poor star-forming regions. Here the CO luminosity defined for *J*=1−0 is obtained with 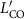
(*J*=1−0)=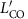
(*J*=2−1) by assuming optically thick and thermalized CO emission. Both the infrared luminosity and SFR are measured after convolution to match the beam of the IRAM 30 m at the CO *J*=2−1 frequency. For comparison, massive star-forming galaxies of approximately solar metallicity^3^,^18^ are also included in the figure. As is well known, the CO luminosity is related to both far-infrared luminosities and SFRs among massive star-forming galaxies, indicating that the molecular gas mass as traced by CO is related to star-formation activities. At a low metallicity, CO decreases because of not only the eliminated reservoir of carbon and oxygen elements but also the increased dissociation of CO molecules by ultraviolet photons under the condition of low dust extinction. As indicated in the figure, both infrared/CO and SFR/CO ratios at a low metallicity are significantly higher than those of massive galaxies. In massive galaxies, infrared luminosity is a good tracer of the SFR, accounting for only a part of the SFR because of the low dust content in metal-poor galaxies. As a result, the increase in the SFR/CO ratio from massive galaxies to metal-poor ones is greater than that in the infrared/CO ratio.

## Discussion

The detection of CO in these objects indicates that molecular gas is present at a very low metallicity. This presence implies that CO can still be a tracer of molecular gas at a very low metallicity. To constrain the conversion factor from the CO luminosity to the molecular gas mass, we estimated the gas mass through the dust emission. All our galaxies have multiband infrared images available in the archive of the Herschel Space Observatory and HI gas maps as observed by Very Large Array^19^. We constructed the infrared spectral energy distribution (SED) of each region covered by the IRAM 30-m beam (11″) and fitted it with a dust model^20^ to derive the dust mass (see Methods section). We used the gas-to-dust ratio of an extremely metal-poor galaxy (Sextans A, 7% solar metallicity)^7^ by assuming the gas-to-dust ratio equal to 8,000(*Z*/0.07)^−1.0^, where *Z* is the metallicity. Here the function of the gas-to-dust ratio with the metallicity is suggested by some observations^21^. Note that we used the same dust model set-up as that for Sextans A to derive the dust mass, thus eliminating the uncertainty caused by the variation of the dust grain properties. After subtracting the atomic gas, the molecular gas mass is obtained. The derived molecular gas has a relatively large uncertainty that results from the photometric error in the infrared SED, the uncertainty in the dust modelling, the HI gas mass error and the error of the gas-to-dust ratio (see Methods section for details).

**Figure 3:**
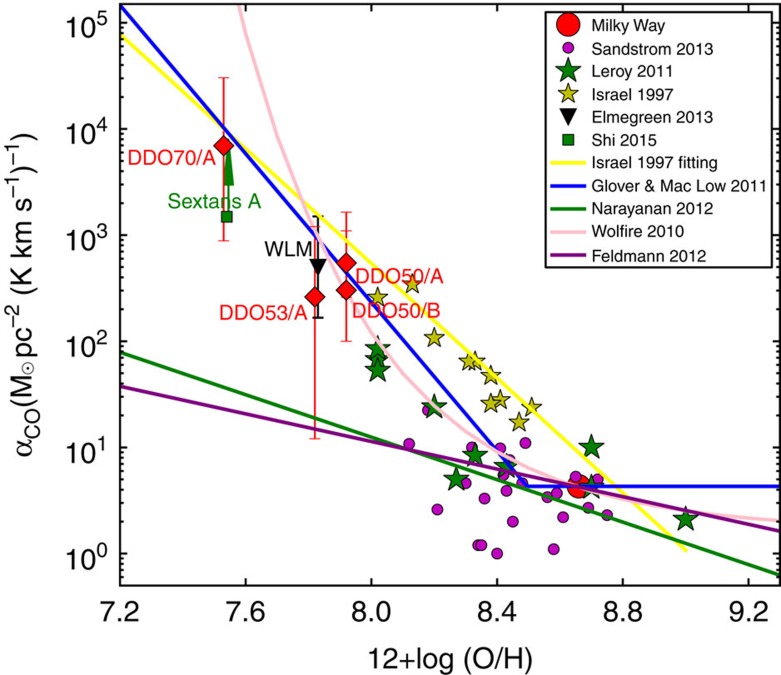
The conversion factor from CO luminosity to molecular gas mass. The four red diamonds denote the result of our metal-poor star-forming regions, and all other symbols denote those observations in the literature^9^,^22^,^23^,^24^. The error bars of our measurements are the s.d., which are caused by the uncertainties on the CO luminosity, the HI gas mass and the dust mass as well as the dust-to-gas ratio. The lines denote models' predictions, including the empirical one^24^ as well as theoretical ones^26^,^27^,^28^,^29^. The parameters in those models were set basically following the literature work^8^, including a linear scaling of the dust-to-gas ratio with the metallicity, and a typical gas surface density of 100 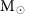 pc^−2^ in the Milky Way.

Figure 3 shows the conversion factor of our metal-poor star-forming regions along with those in the literatures^22^,^23^,^24^,^25^, where the molecular gas content is derived through the spatially resolved dust and HI gas map. Although previous works are limited to the metallicity 12+log(O/H)>8.0, our study indicates that the conversion factor increases rapidly below this metallicity limit. The extremely metal-poor galaxy DDO 70 has a conversion factor about three orders of magnitude higher than the value of the Milky Way galaxy. Another three star-forming regions at 10–20% solar metallicity have conversion factors between ∼100 and 500. One difference in our study compared with those at higher metallicity is that we only targeted the intense star-formation peaks. In these regions, the strong radiation field may increase the effects of CO dissociation, thus biasing the conversion factor towards large values. However, these regions are also infrared peaks with more abundant dust with respective to the rest of the galaxy; such dust may protect CO from dissociation.

In spite of the large uncertainties, the derived conversion factors are still useful to differentiate different theoretical models that give a very large range of predictions at a low metallicity as illustrated in Fig. 3. The empirical relationship (solid yellow line)^24^ based on data above 12+log(O/H)=8.0 is a steep function, and its extrapolations at our metallicity are consistent with our observations. Among all theoretical models, the one that invokes photodissociation of CO and H_2_ self-shielding^26^ matches the observations including ours at a very low metallicity. Other models either overpredict or significantly underpredict the data at the low metallicity end^27^,^28^,^29^.

## Methods

### Observation details

We carried out the CO *J*=2−1 observation using the IRAM 30 m during 22–29 March 2016 (programme ID: 168–15, PI: Y. Shi) with a total of 59.5 h granted. The Eight Mixer Receiver with dual polarization and the Fourier Transform Spectrometers backend were used. To have a good baseline for the spectrum, we adopted the standard wobbler switching mode at a 0.5-Hz beam throwing with an offset of ±120″. The pointing and focussing were set at the beginning of each run and then re-calibrated every 2 h by pointing to the bright quasars close to our targets. The data reduction was performed with CLASS in the GILDAS package. For each region, we averaged all scans to obtain the final one. The effective on-source integration time including two polarization as indicated by CLASS is 1210, 413, 369 and 556 min for DDO70-A, DDO53-A, DDO50-A and DDO 50-B, respectively, with system temperatures of 252, 223, 382 and 283 K, respectively.

Supplementary Fig. 1 shows the CO spectra over a velocity range of ±150 km s^−1^ to illustrate the goodness of a long baseline for our observations. The HI spectrum within each IRAM beam is also extracted from the HI data cube^19^ and overlaid on the CO spectrum as shown in Supplementary Fig. 1. The CO line is within the HI velocity range, although there are some offsets in the central velocity between the two that further validates the reliability of our CO detections.

### Infrared spectral energy distributions

The infrared images from 70 to 250 μm as shown in Supplementary Fig. 2 were retrieved from the archive of the Herschel Space Observatory. The spatial resolutions are about 6″, 12″ and 18″ at 70, 160 and 250 μm, respectively. The data were reduced using the unimap^30^. The standard procedure of the reduction includes the time ordering of the pixels, signal preprocessing, glitch removal, drift removal, making the noise spectrum and generalized least square (GLS) filter, map making with GLS, postprocessing of the GLS map and finally the weighted postprocessing of the GLS map. The mid-infrared images at 3.6, 4.5, 5.8, 8.0 and 24 μm were retrieved from the archive of the Spitzer Space Telescope that is available through the Local Volume Legacy program^31^ with the corresponding spatial resolutions of 1.6″, 1.7″, 1.9″, 2.0″ and 6″, respectively.

To estimate the dust mass, we constructed the infrared SED based on the Spitzer and Herschel images. We first checked the astrometry using field stars and corrected the offsets between the two telescopes, about 1 arcsec for DDO 70 and DDO 53 and 7 arcsec for DDO 50. As the IRAM beam size (11″) is relatively small given the spatial resolutions at those IR wavelengths, the aperture correction is important. We used three approaches to derive the infrared SED. The first approach is to assume point sources for the aperture corrections at all infrared wavelengths and then measure the flux within the IRAM beam for each band at native spatial resolutions. This approach gives the largest possible aperture corrections, which could be treated as upperlimits, given that the star-forming regions are spatially resolved at Spitzer 24 μm and Herschel 70 μm. The following two methods assume that the star-forming regions are extended sources at Spitzer 24 μm and Herschel 70 μm to correct the flux loss for Herschel 160 and 250 μm. For the second approach, we convolved all infrared images above 24 μm to the SPIRE 250 μm using the convolution Kernels^32^ and then measured the flux within the IRAM beam. These fluxes are then corrected for the aperture loss by multiplying with the ratio of the flux of Spitzer 24 μm at its native resolution to that at the convolved resolution. The third method is the same as the second one but convolves the 24 μm, 70 and 160-μm images to Gaussian 11″ beams excluding the 250-μm image, and again, this approach corrects the flux loss with the ratio of the 24-μm flux at the native resolution to that at the convolved resolution. The derived photometric results using three approaches were found to be within 50%. For our discussion, we have adopted the second approach, which adopts images convolved to the Herschel 250 μm resolution and aperture corrections based on the Spitzer 24 μm image, given that the star-forming regions are spatially resolved at 24 μm. As we pointed at the bright infrared peaks, the photon noise is small while the photometric error is instead dominated by the aperture correction because of the small IRAM beam. We assigned 50% of fluxes as systematic uncertainties for the infrared photometry at all wavelengths. The final SED is shown in Supplementary Fig. 3.

### Measurements of the physical properties

Following our previous work^7^, the infrared SED is fitted with a dust model^20^ to obtain the dust mass measurement. We adopted the Milky Way dust grains and fixed the polycyclic aromatic hydrocarbon fraction to be the minimum given the low metallicity of our galaxies. The maximum intensity of the stellar radiation field is further fixed to be 10^6^. Thus the model has three free parameters, including the dust mass, the minimum stellar light intensity and the fraction of dust exposed to the minimum radiation field. An additional 4,000 K black body is included to model the emission from the stellar photosphere. As shown in Supplementary Fig. 3 and listed in Supplementary Table 1, the fitting results are reasonably good. If using the Small Magellanic Cloud (SMC) dust model, the dust mass differs by no more than 15%, which is consistent with previous studies^7^,^33^.

To derive the total gas mass from the dust mass, the gas-to-dust ratio is needed. Unlike our previous work^7^, the infrared observation is not deep enough to derive the gas-to-dust ratio based on the diffuse light for individual galaxies. We thus adopted the gas-to-dust ratio (8,000) of Sextans A at 7% solar from the previous work^7^ that is based on the diffuse light as the value for DDO 70, which is at the same metallicity. Given the large variation in the gas-to-dust ratio from object to object^21^ at this metallicity, we assigned 0.5 dex as 1−*σ* uncertainty of the ratio. For DDO 50 and DDO 53, we assumed a linear increase of the gas-to-dust ratio with the decreasing metallicity following the literature study^21^, with a 1−*σ* uncertainty of ∼0.3 dex at their metallicities. As discussed in the previous work^7^, although different dust grain models provide different dust masses, these different dust masses do not affect the derived gas masses because the dust-to-gas ratio changes accordingly.

To obtain the molecular gas, the atomic gas mass is subtracted from the dust-based total gas mass. The HI gas maps of three galaxies were observed with the Very Large Array through the program of Local Irregulars That Trace Luminosity Extremes^19^. We adopted the robust-weighted maps with the synthesized beam sizes of 13.8″ × 13.2″, 6.3″ × 5.7″ and 7.0″ × 6.1″ for DDO 70, DDO 53 and DDO 50, respectively. Although the DDO 70 has a resolution that is slightly worse than that of our IRAM beam, the HI emission is pretty diffuse so that we can assume the HI mass surface density measured at its resolution is a good approximation of that within the IRAM beam. For DDO 53 and DDO 50, we convolved the HI maps to the 11″ beam to measure the HI mass, which is almost the same (<10%) as those measured at the native resolution, given the HI emission is diffuse. We also retrieved the reduced far-ultraviolet images from the GALEX data archive (http://galex.stsci.edu/GalexView/) whose spatial resolution is about 5″. The SFR is the combination^34^ of the unobscured part (as traced by far-ultraviolet) and the obscured part (as traced by 24-μm emission). The SFRs of massive galaxies used for comparison in Fig. 2 are based on their infrared luminosities^3^ using the formula^35^ with corrections for Chabier initial mass function (IMF). The stellar mass is derived based on the 3.6 and 4.5-μm emission^36^.

### Data availability

The data that support the findings of this study are available from the corresponding author upon reasonable request.

## Additional information

**How to cite this article:** Shi, Y. *et al*. Carbon monoxide in an extremely metal-poor galaxy. *Nat. Commun.* **7**, 13789 doi: 10.1038/ncomms13789 (2016).

**Publisher's note:** Springer Nature remains neutral with regard to jurisdictional claims in published maps and institutional affiliations.

## Supplementary Material

Supplementary Information Supplementary Figures 1-3 and Supplementary Table 1

## Figures and Tables

**Table 1:**
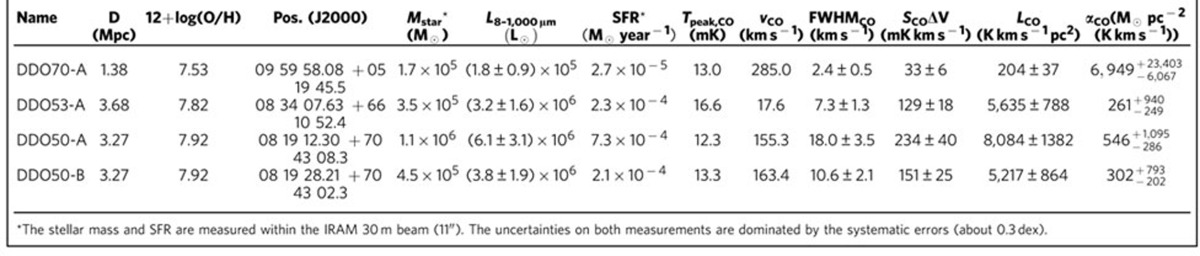
Properties of IRAM-30m-targeted regions.
