# Supplementary material for: Carbon monoxide in an extremely metal-poor galaxy
Source: Nat Commun. 2016 Dec 9;7:13789. doi: 10.1038/ncomms13789 (PMC5155163; doi:10.1038/ncomms13789)
Supplement: Supplementary Information — Supplementary Figures 1-3 and Supplementary Table 1 [file ncomms13789-s1.pdf]

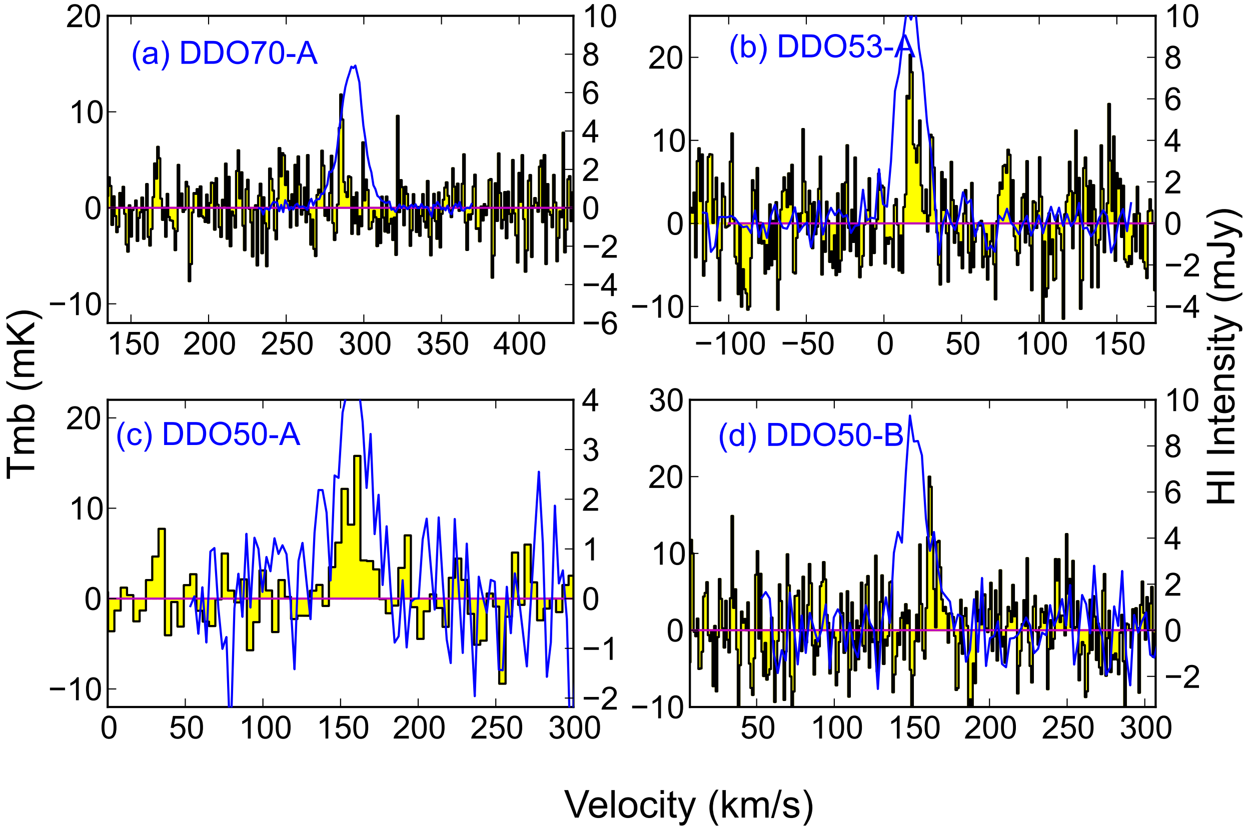

Supplementary Figure 1 | The CO J=2-1 spectra (yellow area) overlaid with the HI spectra (blue lines). The whole velocity range is +/- 150 km/s.

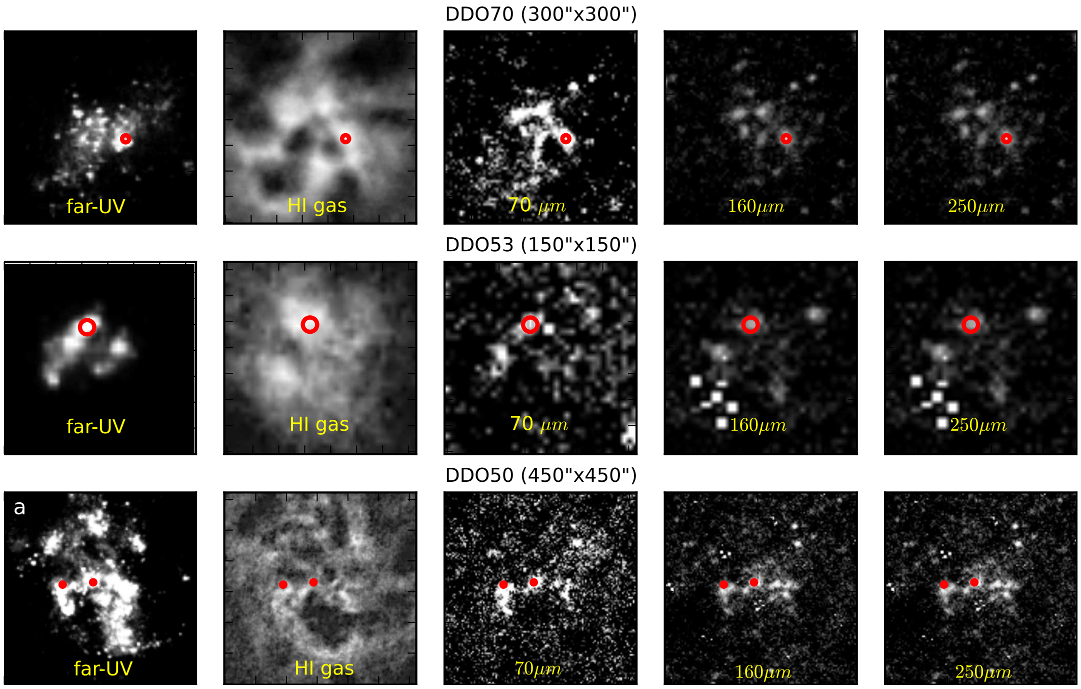

Supplementary Figure 2 | The multi-wavelength image of galaxies: from left to right, the far-UV, HI atomic gas, 70 μm, 160 μm and 250 μm images. The red circle in each panel indicates the IRAM beam size.

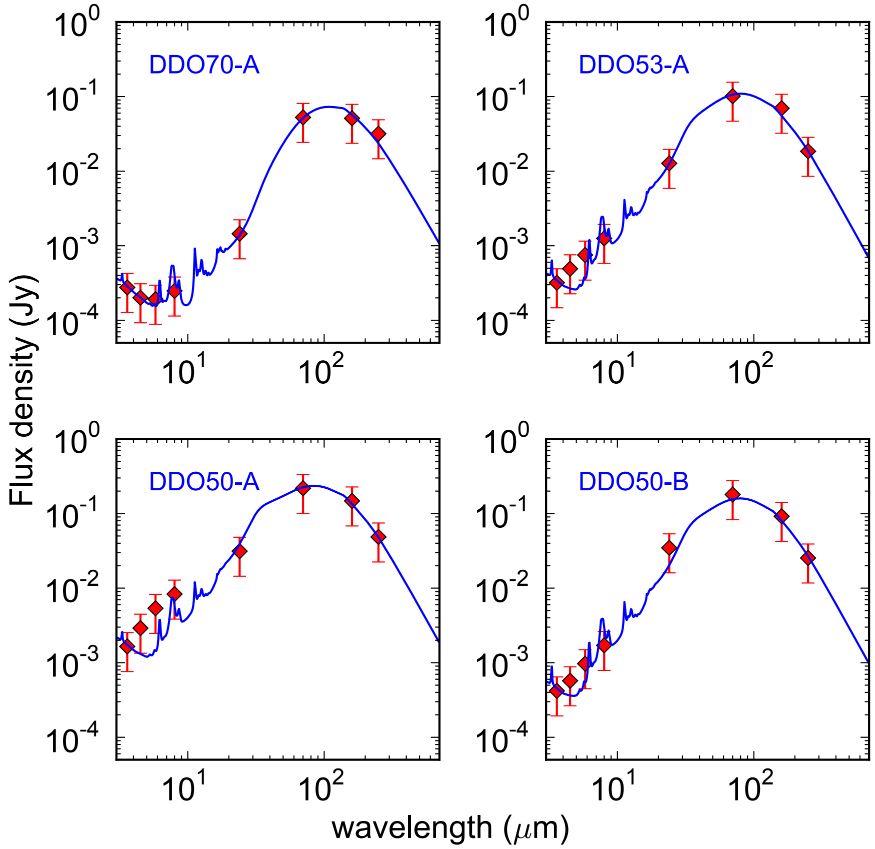

Supplementary Figure 3 | The infrared SED of each IRAM-30m pointing region: the symbols denote the observed photometry with the error bars defined as the standard deviation. The solid line is the best-fitted dust model.

Supplementary Table 1 The infrared photometry and SED fittings

name	f(3.6μm) (mJy)	f(4.5μm) (mJy)	f(5.6μm) (mJy)	f(8.0μm) (mJy)	f(24μm) (mJy)	f(70μm) (mJy)	f(160μm) (mJy)	f(250μm) (mJy)	χ^2/d.o.f.	$M_{\rm dust}$ ($M_\odot$)
DDO70/A	0.28$\pm$0.138	0.20$\pm$0.100	0.19$\pm$0.096	0.25$\pm$0.12	1.45$\pm$0.72	52$\pm$ 26	51$\pm$ 25	31$\pm$ 15	0.37	$(1.9^{+1.5}_{-0.9})$x10^2
DDO53/A	0.32$\pm$0.159	0.49$\pm$0.245	0.75$\pm$0.375	1.25$\pm$0.63	12.72$\pm$6.36	101$\pm$ 50	69$\pm$ 34	18$\pm$ 9	0.19	$(6.1^{+1.6}_{-1.2})$x10^2
DDO50/A	1.65$\pm$0.825	2.91$\pm$1.453	5.37$\pm$2.683	8.32$\pm$4.16	31.29$\pm$15.65	218$\pm$ 109	147$\pm$ 73	48$\pm$ 24	1.12	$(1.5^{+0.5}_{-0.3})$x10^3
DDO50/B	0.42$\pm$0.209	0.57$\pm$0.286	0.97$\pm$0.485	1.71$\pm$0.85	34.70$\pm$17.35	179$\pm$ 89	91$\pm$ 45	25$\pm$ 12	1.24	$(7.^{+1.2}_{-0.9})$x10^2

The photometric error is dominated by the systematic uncertainty that is set to be 50% of the flux as detailed in the text.
